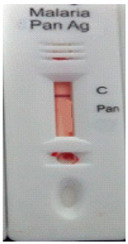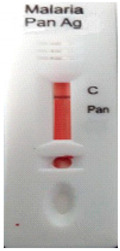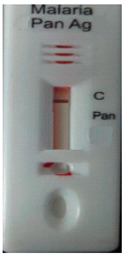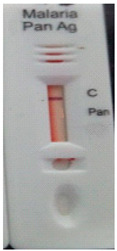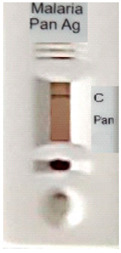# Optical Density Optimization of Malaria Pan Rapid Diagnostic Test Strips for Improved Test Zone Band Intensity

**DOI:** 10.3390/diagnostics10110880

**Published:** 2020-10-29

**Authors:** Prince Manta, Rupak Nagraik, Avinash Sharma, Akshay Kumar, Pritt Verma, Shravan Kumar Paswan, Dmitry O. Bokov, Juber Dastagir Shaikh, Roopvir Kaur, Ana Francesca Vommaro Leite, Silas Jose Braz Filho, Nimisha Shiwalkar, Purnadeo Persaud, Deepak N. Kapoor

**Affiliations:** 1 School of Pharmaceutical Sciences, Shoolini University of Biotechnology and Management Sciences, Solan 173212, India; princemanta@gmail.com; 2 School of Bioengineering and Food Technology, Shoolini University of Biotechnology and Management Sciences, Solan 173212, India; rupak.nagraik@gmail.com (R.N.); avinashsubms@gmail.com (A.S.); 3 Department of Surgery, Medanta Hospital, Gurugram 122001, India; drakshay82@gmail.com; 4 Departments of Pharmacology, CSIR-National Botanical Research Institute, Lucknow 226001, India; preetverma06@gmail.com (P.V.); paswanshravan@gmail.com (S.K.P.); 5 Institute of Pharmacy, Sechenov First Moscow State Medical University,8 Trubetskaya St., Moscow 119991, Russia; bokov_d_o@staff.sechenov.ru; 6 Department of Neurology, MGM Newbombay Hospital, Vashi, Navi Mumbai 400703, India; jubershaikh703@yahoo.com; 7 Department of Anesthesiology, Government Medical College, Amritsar 143001, India; roopvirsaini@gmail.com; 8 Department of Medicine, University of Minas Gerais, Passos 37902-313, Brazil; francescavommaroleite@gmail.com (A.F.V.L.); silasbrazf@gmail.com (S.J.B.F.); 9 Department of Anesthesiology, MGM Hospital, Navi Mumbai 410209, India; dr.nimisha4u@gmail.com; 10 Department of Medicine, Kansas City University, Kansas City, MO 64106, USA; narpaulpersaud@hotmail.com

**Keywords:** lateral flow assay, immuno-chromatographic, gold nanoparticles sensor, UV/Vis spectrophotometer, malaria pan rapid diagnostic strip, point-of-care

## Abstract

For the last few decades, the immunochromatographic assay has been used for the rapid detection of biological markers in infectious diseases in humans and animals The assay, also known as lateral flow assay, is utilized for the detection of antigen or antibody in human infectious diseases. There are a series of steps involved in the development of these immuno-chromatographic test kits, from gold nano colloids preparation to nitrocellulose membrane coating (NCM). These tests are mostly used for qualitative assays by a visual interpretation of results. For the interpretation of the results, the color intensity of the test zone is therefore very significant. Herein, the study was performed on a malaria antigen test kit. Several studies have reported the use of gold nanoparticles (AuNPs) with varying diameters and its binding with various concentrations of protein in order to optimize tests. However, none of these studies have reported how to fix (improve) test zone band intensity (color), if different sized AuNPs were synthesized during a reaction and when conjugated equally with same amount of protein. Herein, different AuNPs with average diameter ranging from 10 nm to 50 nm were prepared and conjugated equally with protein concentration of 150 µg/mL with K_D_ = 1.0 × 10^−3^. Afterwards, the developed kits’ test zone band intensity for all different sizes AuNPs was fixed to the same band level (high) by utilization of an ultraviolet-visible spectrophotometer. The study found that the same optical density (OD) has the same test zone band intensity irrespective of AuNP size. This study also illustrates the use of absorption maxima (λ max) techniques to characterize AuNPs and to prevent wastage of protein while developing immunochromatographic test kits.

## 1. Introduction

Malaria is caused by parasites that are transmitted to humans via the bites of the infected female Anopheles mosquito. While preventable and curable, it still remains a paramount cause of morbidity and mortality in developing countries. Malaria is estimated to kill between 1.5 to 2.7 million people annually [1]. Malaria morbidity is estimated at about 300–500 million annually, and malaria clinical diagnosis is most effective at 50%. Malaria immunoassays use the inherent sensitivity, specificity and binding affinity of antibodies to respective antigens for the detection of antigens in a sample. In immunoassays, the sample tested includes whole blood, urine, saliva, serum, etc. [2]. In the Malaria Pan Antigen rapid test kit, the sample used is Red Blood cells containing specific antigens of *P. vivex and P. malariae/P. ovale* [3]. The red blood cells get lysed by a buffer solution to allow antigen–antibody binding at the test site. Immunoassay signals emanate from the gold-labeled antibody set for the antigen on a substratum at the binding site (Test line). Typical antibody labels include fluorescent molecules, nano- or microparticles, or enzymes. Gold nanoparticles (NPs) are the most widely used label [4]. Such immunoassays can be used in industry, clinical or laboratory settings, doctor’s offices, or as over-the-counter tests [2]. At the test line, the naked eye will see a gold-labelled marker as a pink/red line [5]. In most countries, the diagnosis of malaria challenges multiple laboratories [3]. The laboratories require longer than one hour to analyze the findings, leading to less consistency in the analysis of the results.

### 1.1. Components of Immuno-Chromatographic Test Kits

**Figure 1:**
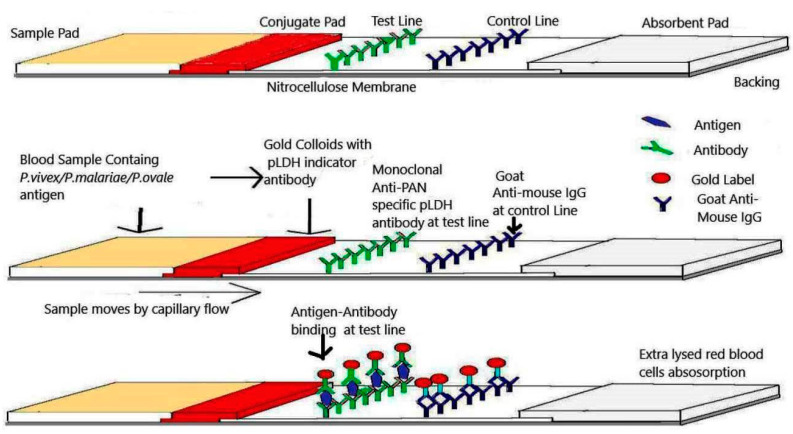
Presentation of lateral flow strip that works on sandwich assay. Blood sample lysed with buffer solution is added to the sample pad. *P. vivex/P. malariae/P. ovale* malaria antigens attach to antibodies in the red colored gold conjugate pad and the complex formed attaches to test line monoclonal anti-PAN specific pLDH antibodies. The excess labeled antibodies bind with Goat anti-mouse IgG antibodies in the control line. The extra lysed red blood cells get absorbed in the absorbent pad.

The Immuno-Chromatographic kit is composed of components shown in Figure 1. The parts of the kits are attached on an inert polyvinyl chloride (PVC) backing material and further packed in a plastic cassette with a specimen port and reaction window displaying the capture and control zones [2]. The Immunochromatographic Test Kit has a sample pad, conventionally composed of glass fibres. The sample pad is selected to have zero cross-reactivity with the specimen. The sample pad is pretreated with a buffer for specimen pH adjustment and extraction of unspecific antigen form specimens [6]. One of the vital parts of the strip is nitrocellulose membrane (NCM). In this, the interaction between antigen and antibody takes place. Typically, a hydrophobic nitrocellulose membrane is used on which anti-target analyte antibodies are immobilized in a line that crosses the membrane to act as a capture zone on the test line [2]. The NCM membrane should be chosen based upon pore size [7]. Other parts of test strips are glass fibres or non-woven fibres based conjugate pads which can be pre-treated to avoid any cross-reactivity [8]. Conclusively, the conjugate pad is prepared by dipping the glass fibers into a colloidal solution of gold protein and then used after drying. In addition, an absorbent pad is present in the kit, which is designed to collect extra specimen samples passing the reaction membrane [9].

### 1.2. The Protein

In the Malaria Pan immunoassay, antibody protein is used for AuNP conjugation. Plasmodium lactate dehydrogenase (pLDH) and goat anti-mouse (GAM) protein are used for binding at test and control lines, respectively. An ultraviolet-visible spectrophotometer optimization technique was demonstrated in this work by formulating an immuno-chromatographic detection kit for Malaria Pan using AuNPs as an indicator. Various research works attempted to optimize the AuNP size [10,11,12,13], and the AuNPs of about 30–40 nm were reported to be optimal [11,12]. Khlebtsov and Byzova et al. also tried to determine the optimum concentration of protein required for AuNP conjugation [14,15].

In present research, gold nanoparticles (AuNPs) were utilized as labels, and the concentration of AuNPs with conjugate antibodies was tailored to a fine-tuned optical density (OD). The gold nanoparticles of various sizes (10 nm to 50 nm) were prepared, by quantifying λ max (absorption maxima) and dynamic light scattering (DLS). The relationship of AuNP diameters with a concentration of target protein was monitored to develop a better test kit. Finally, the developed immuno-chromatographic test kit test zone band intensity was tested using RGB and HSV color models. The reason to select a malaria test kit for the study is to create a more cost-effective rapid diagnostic test kits because malaria cases are found in countries where cost-effectiveness is significant. The study aim to improve test band intensity irrespective of AuNP size using a fixed quantity of protein while optimizing the optical density.

## 2. Materials and Methods

### 2.1. Reagents, Instruments and Other Support Materials

For the fabrication of immunochromatographic strip assay, sodium hydrogen phosphate, sucrose, disodium hydrogen phosphate, sodium chloride and bovine serum albumin (BSA) were purchased from Merck, Darmstadt, Germany. The gold chloride used for the synthesis of gold nanoparticles was purchased from Sigma-Aldrich, Tokyo, Japan. The Plasmodium lactate dehydrogenase (pLDH) antibodies’ molecules and control line Goat anti-mouse protein were purchased from Fapon Biotech, Shenzhen, China. All the other chemicals and reagents used in the present study were of analytical grade reagents. The Delsa™ Nano Submicron Particle Size Zeta Potential instrument of Beckman Coulter, Brea, CA, USA was used for analyzing AuNP diameters. An ultraviolet-visible spectrophotometer 1900i of Shimadzu, Kyoto, Japan was used to measure optical density and absorbance maxima. The nitro cellulose membrane was coated with XYXYZ3210™ dispense platform of Bio-dot, Irvine, CA, USA. The centrifuge of Remi RM-12C, Mumbai, India was utilized for AuNP–protein conjugate centrifugation, and the magnetic stirrer of Remi, Mumbai, India was also used in the study. The nitrocellulose membrane was purchased form Nupore System Pvt. Ltd., Ghaziabad, India and Glass fibre sample pad and conjugate pad were purchased from Advanced Micro Devices, Ambala, India.

### 2.2. Experimental

#### 2.2.1. Preparation of Gold Nanoparticles (AuNPs)

The gold nanoparticles were prepared by classical classical Turkevich and Fern methods by citrate reduction. In general, the Turkevich and Fern process reaction leads to formation of AuNPs of size range 10 nm to 100 nm [16,17]. Utilizing raw gold chloride (AuHCl_4_) [18], the 1% light yellowish color solution was prepared by dissolving 1 gm of gold chloride in 100 mL of ultra-mili-Q water. The aforementioned 1% gold solution was furthermore dissolved into ultra-milli-Q water in order to obtain the optical density between 0.7 and 0.9 at λ max (absorption maxima) by taking a solution spectrum scan at wavelength between 700 nm and 400 nm. Now, the final gold solution had been refluxed for 30 min at 100 °C. The above-diluted gold solution was reduced by adding 1% (1 g of sodium citrate in 100 mL of water) sodium citrate solution of pH 7.80 ± 0.5 with refluxing until bright red color develops. Initially, the addition of a 1% solution of sodium citrate turns the color of the solution black. The color change from mildly yellowish to brick red confirms the synthesis of nanoparticles. The change in colour solution is due to the surface plasmon resonance effect (SPR) in which electrons excited to its higher state and produces a colour change. During the reduction mechanism, metal salts get converted into their ionic form when it combines with water. Different chemical functional groups of reducing agents combine with metal ions whether they are bivalent or monovalent and reduce it into a zerovalent state of small size [19].

The color transforms from red to pink to blue as the reaction proceeds. As the solution color turns pink, the reaction was stopped by decreasing the temperature to room temperature in the ice bath. Particle size distribution of synthesized nanoparticles was analysed by dynamic light scattering.

The five gold nanoparticles of the sizes 10 nm, 20 nm, 30 nm, 40 nm and 50 nm were chosen for protein conjugation after AuNP characterisation. The prepared pink-colored gold solution was characterized by spectrophotometric absorbance maxima (λ max) by scanning in a visible wavelength range of 700 nm to 400 nm.

#### 2.2.2. Protein Conjugation with Gold NPs

For all the above-prepared AuNPs of size 10 nm to 50 nm, pH was adjusted separately to 7.00 ± 0.1 with 0.2 M Potassium Carbonate solution of pH 12.00 ± 0.5. The pH adjustment is predicated on the protein’s isoelectric point, which varies from protein to protein. The pH was measured with the help of pH paper. The antibody pLDH (Plasmodium lactate dehydrogenase) reagents have been diluted to 150 μg/mL from the stock solution with 10 mM of Sodium Dihydrogen Phosphate buffer of pH 8.50 ± 0.1. Afterwards, the protein pLDH of 150 μg/mL concentration was conjugated to all five AuNPs (10 nm to 50 nm). Conjugation of the AuNPs and protein was achieved by stirring the solution to 10 ± 2 min. Following this, 1% BSA (Bovine Serum Albumin) was added into the gold conjugate solution and stirred for 30 ± 2 min for stabilisation and abstraction of unbound protein. For all five sizes of AuNPs, the single tuned protein concentration was used to detect the effect of gold nanoparticle size on the band intensity of developed kits.

#### 2.2.3. Centrifugation

**Figure 2:**
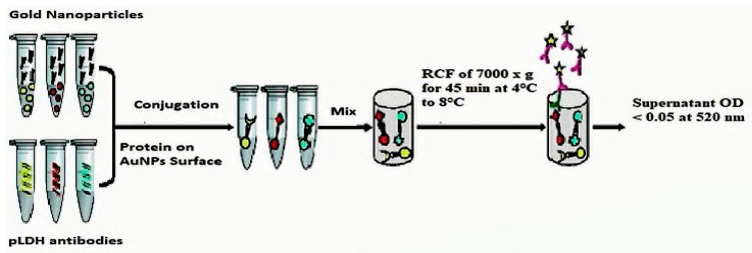
The diagrammatical representation of the protein conjugation and centrifugation methodology.

The above five separately prepared AuNP–protein conjugate solutions were centrifuged. The centrifugation was performed with a Relative Centrifugal Force (RCF) of 7000× *g* for 45 min at 4 °C to 8 °C temperature. The centrifugation of an AuNPs–protein conjugate solution at a force higher than 7000× *g* RCF can sometimes shows aggregation while centrifugation at a force slower than 7000× *g* RCF may give less residue with a dark supernatant. Centrifugation at 7000× *g* RCF gives a stable and good yield of the residue or pellet. The supernatant’s aspiration was performed in a different beaker, and gold pellets were resuspended in the phosphate-buffered saline (PBS) buffer. The absorbance of the supernatant was measured at a 520 nm wavelength. If the OD was greater than 0.05, then the supernatant’s re-centrifugation is performed one more time. The supernatant was discarded if the OD was less than 0.05. This will enable conjugate recovery and prevent wastage. The supernatant aspiration is performed in a separate beaker, accumulating the AnNP–protein pellets. Carefully, the supernatant aspiration and the residue re-suspension was accomplished in a re-suspension buffer. Figure 2 represents the protein conjugation and centrifugation procedure.

#### 2.2.4. Conjugate Pad Prepration

All five separately re-suspended AuNPs—protein conjugate above solutions were diluted (ultra-pure mili-Q water) to a constant OD of 3.00 at 520 nm wavelength.

Following the dilution, five conjugate pads were prepared by dipping the glass fibre pad into the conjugate solution. In comparison, the other changes through the entire development of the kit was held constant, e.g., test line concentration and control line protein.

#### 2.2.5. Membrane Coating

The five nitrocellulose membranes were coated at the test (Pan) and control line (C). The test and control line coating on the nitrocellulose membrane (NCM) was achieved with the use of a Bio-dot dispensing machine. First of all, the bio-dot machine stripping system (tubing and jets) was flushed with de-ionized water over ten cycles. The control and test solutions were then coated on NCMs. For drying, membrane sheets were kept in the oven at 30 °C for 30 min after coating. The concentrations of test and control line reagents were as follows:

#### 2.2.6. Test Line Reagents Concentration

To obtain the final test solution, the pLDH (Plasmodium lactate dehydrogenase) antibody was diluted from the stock solution to 50 μg/mL with 1% sucrose solution in the PBS buffer. The antibody protein mixing in PBS buffer was performed with a magnetic stirrer, and a 0.22-micron filter was used to eliminate the suspended particles.

#### 2.2.7. Control Line Reagents Concentration

To obtain the final control line solution, Goat Anti Mouse IgG was diluted from stock solution to 400 μg/mL with a 0.5 percent sucrose in the PBS buffer. To extract the suspended particles, mixing and filtration were achieved using a 0.22-micron filter.

## 3. Results and Discussion

### 3.1. Gold NP Characterization

**Figure 3:**
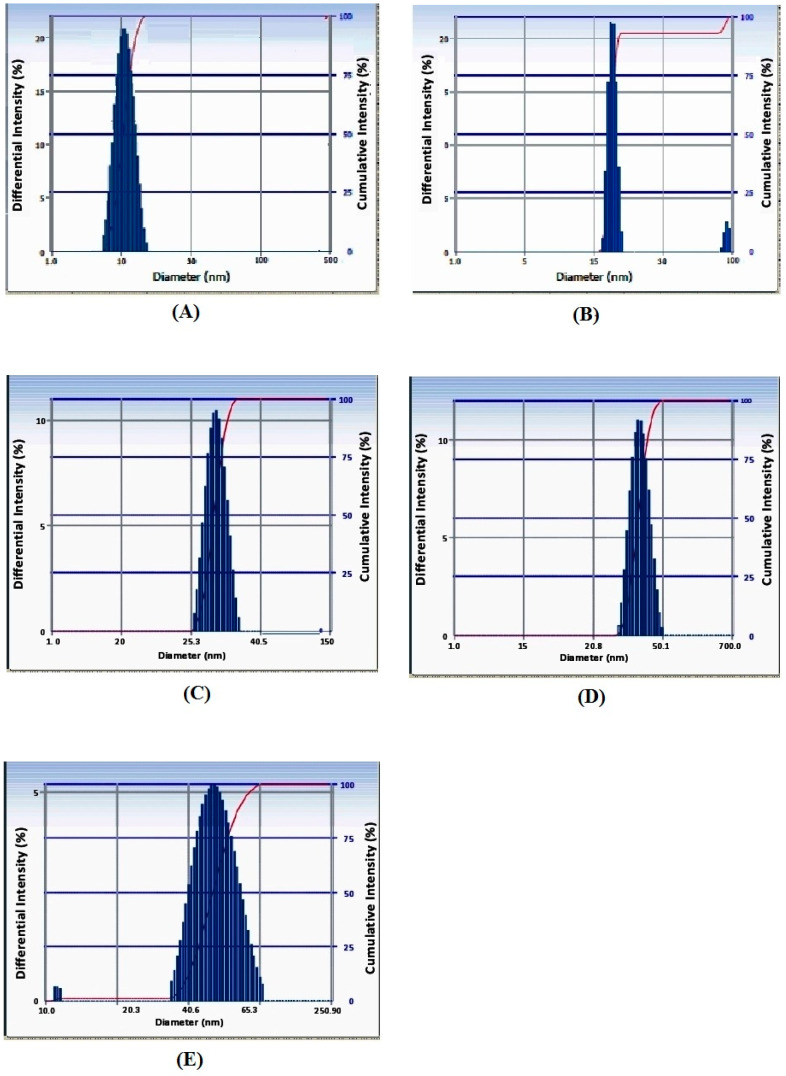
Synthesized gold nanoparticles’ size distribution measured by the Zeta Seizer. The sodium citrate solutions of 0.2, 0.4, 0.6, 0.75 and 0.90 mg/mL in gold solutions’ produced nanoparticles (NPs) with average diameters of (**A**) 10 nm (size distribution of 8 to 12 nm); (**B**) 20 nm (size distribution of 17 to 23 nm); (**C**) 30 nm (size distribution of 26 to 35 nm); (**D**) 40 nm (size distribution of 32 to 50 nm); and (**E**) 50 nm (size distribution of 36 to 80 nm).

**Figure 4:**
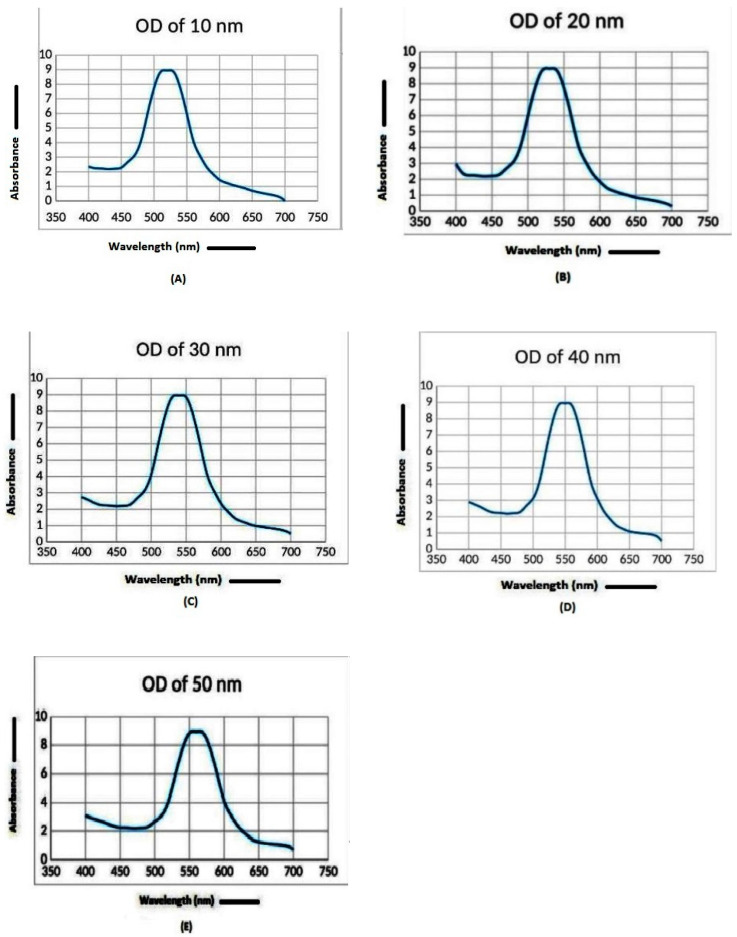
Optical density spectrum of synthesized gold nanoparticles. The gold nanoparticles (AUNPs) show different λ max (absorbance maxima) at different sizes; i.e., (**A**) λ max 520 nm at diameter of 10 nm; (**B**) λ max 530 nm at diameter of 20 nm; (**C**) λ max 540 nm at diameter of 30 nm; (**D**) λ max 560 nm at diameter of 40 nm; (**E**) λ max 570 nm at diameter of 50 nm.

**Figure 5:**
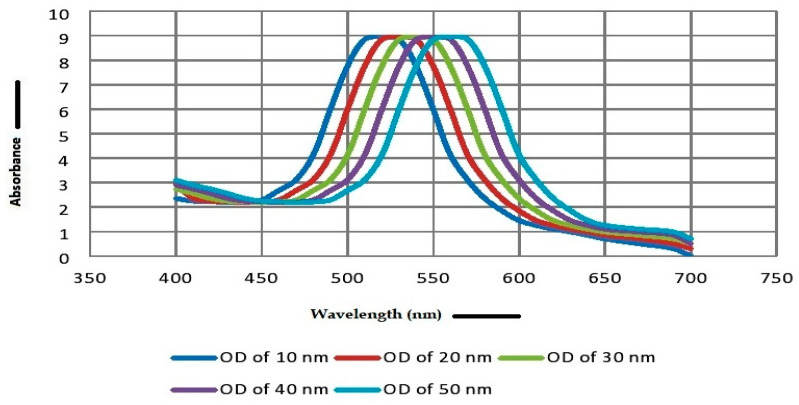
The graph shows a correlation of synthesized gold nanoparticles (10 nm, 20 nm, 30 nm, 40 nm and 50 nm) λ max (absorbance maxima) and Optical Density. With an increase in the size of the gold NPs, the absorbance maxima (λ max) shifts to higher wavelengths (520 nm to 570 nm), reflecting nanoparticle instability.

Firstly, we prepared the most stable AuNPs of an average of of 10 nm to 50 nm in diameter. The AuNPs with absorbance maxima (λ max) ranging between 520 to 570 wavelengths were considered for the development of the Malaria Pan Antigen detection test kit. In this range of λ max, the AuNP size ranges from 10 nm to 50 nm as determined by the particle sizer. Figure 3A–E represent the AuNP size measured in dynamic light scattering (DLS). AuNPs of this range were selected due to their smaller particle size and lower polydispersity index (PDI). It has been observed that smaller sized nanoparticles have better conjugation with the protein [10,11,12,20]. The size of gold NPs depends on the sodium citrate content used [18]. The concentration of sodium citrate in gold solution affects the size of AuNPs, which can be controlled by measuring absorbance maxima (λ max). The sodium citrate of viz 0.2, 0.4, 0.6, 0.75 and 0.90 mg/mL in gold solutions produces nanoparticles (NPs) with average diameters of 10 nm (size distribution of 8 to 12 nm), 20 nm (size distribution of 17 to 23 nm), 30 nm (size distribution of 26 to 35 nm), 40 nm (size distribution of 32 to 50 nm) and 50 nm (size distribution of 36 to 80 nm), respectively (Figure 3). AuNPs shows λ max at varying wavelengths of 520 (10 nm), 530 (20 nm), 540 (30 nm), 560 (40 nm) and 570 (60 nm). Figure 4A–E represent the λ max of AuNPs. The optical density of prepared AuNPs at λ max ranges between 8.0 to 9.0. When sodium citrate concentration increases in AuNP solution, AuNP size does too. When the size of the gold NPs increases, the absorbance maxima (λ max) shift to higher wavelengths (Figure 5), and the color of the solution turns from pink to blue, reflecting nanoparticles’ instability.

As the average diameters increase, the nanoparticles’ size distribution increases, which causes the instability of AuNPs (Figure 3). This technique is widely used for the determination of particle size in colloidal solution, which, in turn, used to measure the thickness of capping or stabilizing agent along with its actual size of metallic core. These studies also determined the hydrodynamic diameter of the synthesized nanoparticles. These results also suggested that there is an absence of large aggregates when these nanoparticles were dispersed in aqueous medium [21].

### 3.2. Monitoring the Protein Loss

After centrifigation of AuNP–protein conjugate, a small portion of re-suspended solution was diluted (1 in 100) into ultra-pure mili-Q water to facilitate OD measurement at 520 nm. The OD values obtained are shown in Table 1.

**Figure 6:**
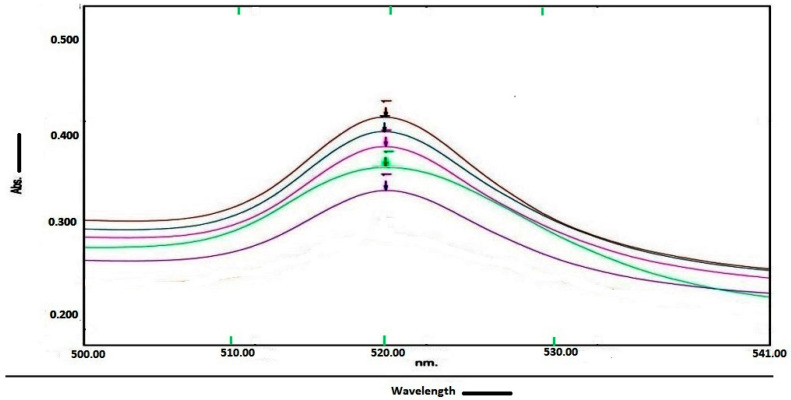
The spectrum overlay graph shows optical density of gold nanoparticles (10 nm, 20 nm, 30 nm, 40 nm and 50 nm) and protein conjugate solution (1–100 mL) measured at 520 nm of wavelength. The gold nanoparticles of average diameter of 40 nm have maximum optical density (0.426), which reflects the maximum protein binding.

Figure 6 and Table 1 indicate that 40 nm AuNPs have high OD, and thus AuNPs have maximum protein binding with AuNPs of 40 nm size. In contrast, the other AuNPs had less binding. This indicates that the supernatant lost extra unbound protein. This means that the additional unbound protein was lost in the supernatant. The protein depletion can also be checked with the use of UV/Vis spectrophotometer OD analysis.

### 3.3. Test Line Intensity Analysis

**Figure 7:**
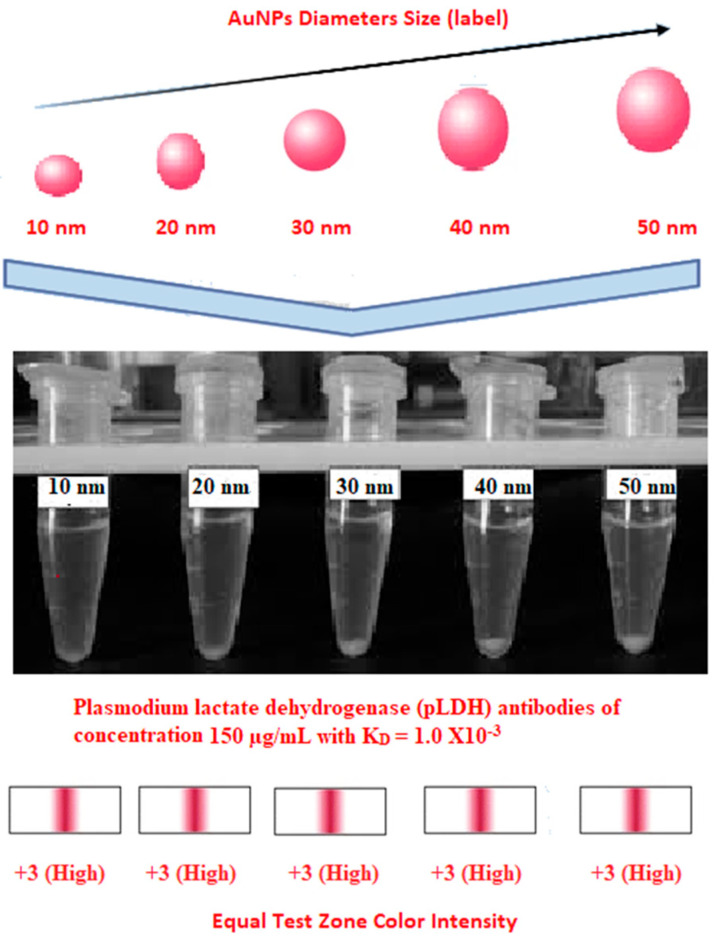
The diagram systematically represented the results.

The developed kits were tested to find out the kit test zone band intensity (results) when equivalent protein ratios are conjugated with different AuNP sizes. The five immunochromatographic rapid test kits were formulated using a conjugate pad (10 nm to 50 nm) prepared above. Then, all five of the immunechromatographic test kits were assembled. Now, the five developed test kits were tested for band intensity using 5 μL of malaria (*P. vivax*) positive blood specimens of concentration 150 parasites/μL. The three specifications were given to test line viz. high test line intensity was ranked as +3, medium test line intensity was ranked as +2, and weak test line intensity was ranked as +1. Upon testing all kits with the same specimen samples, all kits showed an equal band intensity of +3 (high) as shown in Table 2. In this analysis, it was noticed that, if the final OD is tuned to one point, there will be no effects of AuNPs sizes on kit results (test zone intensity). OD-adjustment will refine the final test zone band intensity. Figure 7 is the systematic representation of the results. All five test kits developed after OD tuning to 3.0 were additionally verified for their specificity with 5 μL of Malaria Pf (Falciparum) antigen blood specimens of concentration 40 parasites/μL to find out test kit susceptibility for *P. Falciparum*. The specificity results of developed kits (Table 3) were found without any false positive indication (no Pan line appears), and the the control band intensity was high (+3).

## 4. Conclusions

It can be concluded from the above study that the particle size of AuNPs has no effect on the test zone band intensity of Malaria Pan rapid diagnostic test kits if optical density of AuNP–protein conjugate is adjusted at 520 nm. The test zone band intensity was also observed to be maximum at 520 nm and optical density 3.0. It was found from the study that any quantity of protein can be utilized for AuNP conjugation, if the final optical density (OD) is adjusted correctly. It can also be concluded that, by optimizing the optical density, an enhanced test zone band intensity can be obtained while reducing the total number of trial and wastage of reagents.

## Figures and Tables

**Table 1:** Optical density (OD) measurement results of synthesized gold nanoparticles–protein centrifuged re-suspension conjugate solution when diluted in 1–100.

S. No.	Gold Nanoparticles (AuNP) Size	Optical Density (OD) Observation
1	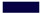 10 nm	0.301
2	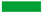 20 nm	0.354
3	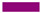 30 nm	0.368
4	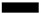 40 nm	0.426
5	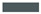 50 nm	0.385

**Table 2:** Test line intensity results of five Malaria Pan Ag immunochromatographic rapid test kits formulated using different diameter AuNPs. The kit final OD is tuned to one point (3.00) after conjugation with protein. Kit’s test line intensity was tested using *P. vivax* positive blood specimen of a concentration of 150 parasites/μL. The band intensity for test (Pan) and control (C) line is ranked as: high test (+3), medium (+2) and weak (+1). Upon testing, all developed kits showed an equal sensitivity of +3 (high).

Test Zone Intensity of Kit Developed by 10 nm of AuNPs	Test Zone Intensity of Kit Developed by 20 nm of AuNPs	Test Zone Intensity of Kit Developed by 30 nm of AuNPs	Test Zone Intensity of Kit Developed by 40 nm of AuNPs	Test Zone Intensity of Kit Developed by 50 nm of AuNPs
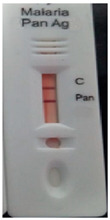	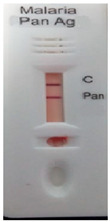	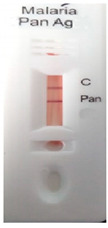	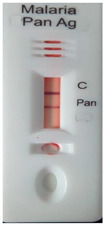	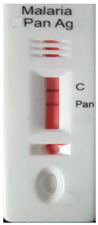
+3 (High) Intensity	+3 (High) Intensity	+3 (High) Intensity	+3 (High) Intensity	+3 (High) Intensity

**Table 3:** Specificity results of five Malaria Pan Ag immunochromatographic rapid test kits formulated using different sizes AuNPs. Kit’s specificity was tested using *P. Falciparum* positive blood specimens of concentration 40 parasites/μL. The color band intensity for control (C) line was +3 (high) and no line appears on the test zone (Pan) indicating absence of any cross reactivity.

Specificity of Kit Developed by 10 nm of AuNPs	Specificity of Kit Developed by 20 nm of AuNPs	Specificity of Kit Developed by 30 nm of AuNPs	Specificity of Kit Developed by 40 nm of AuNPs	Specificity of Kit Developed by 50 nm of AuNPs